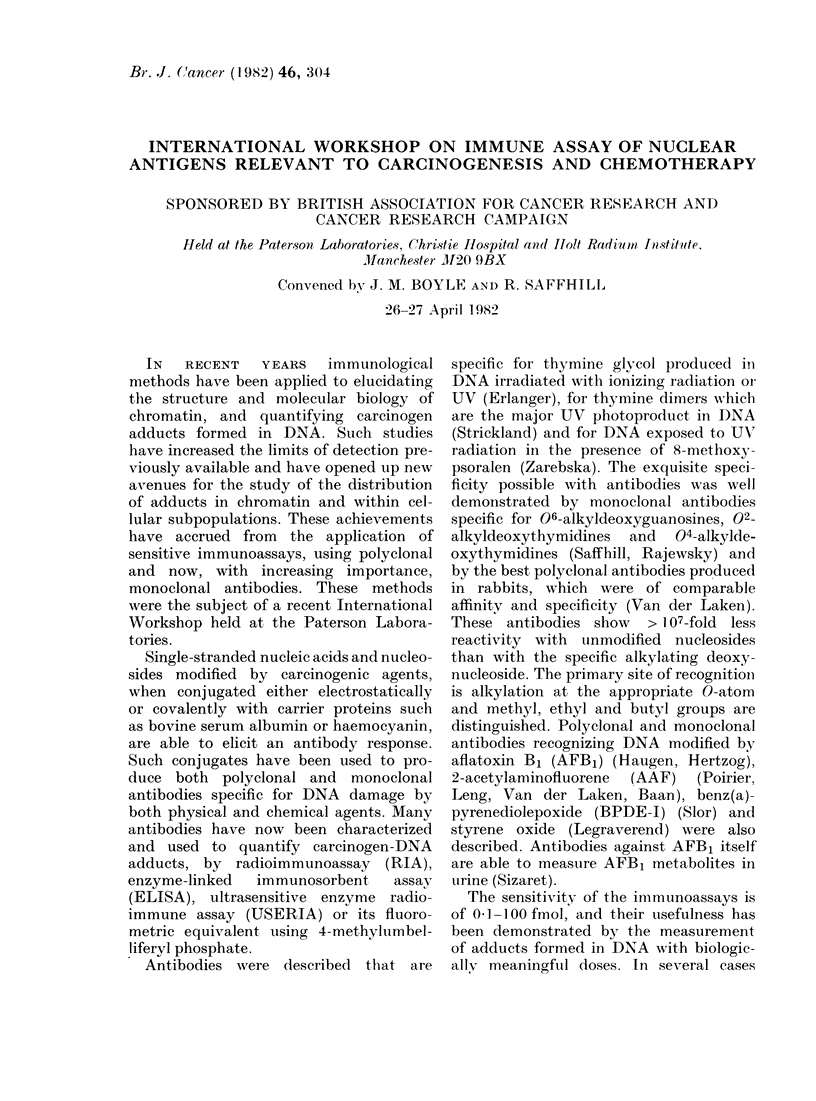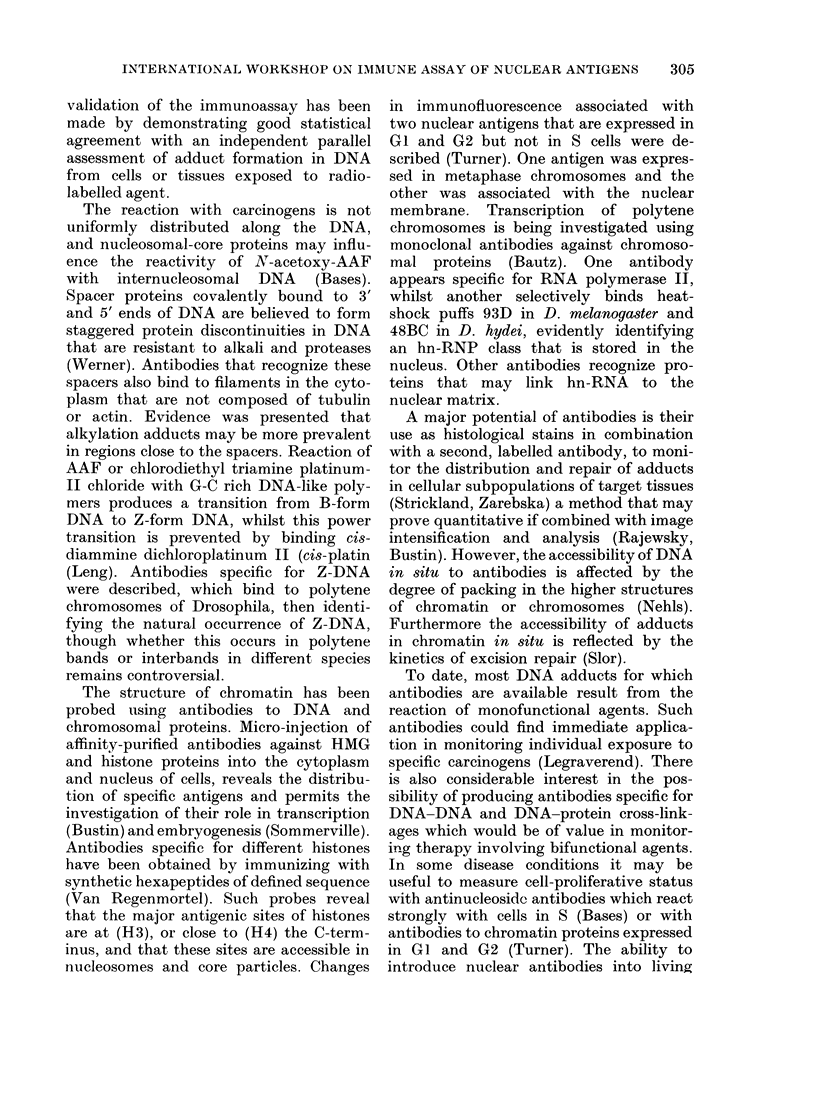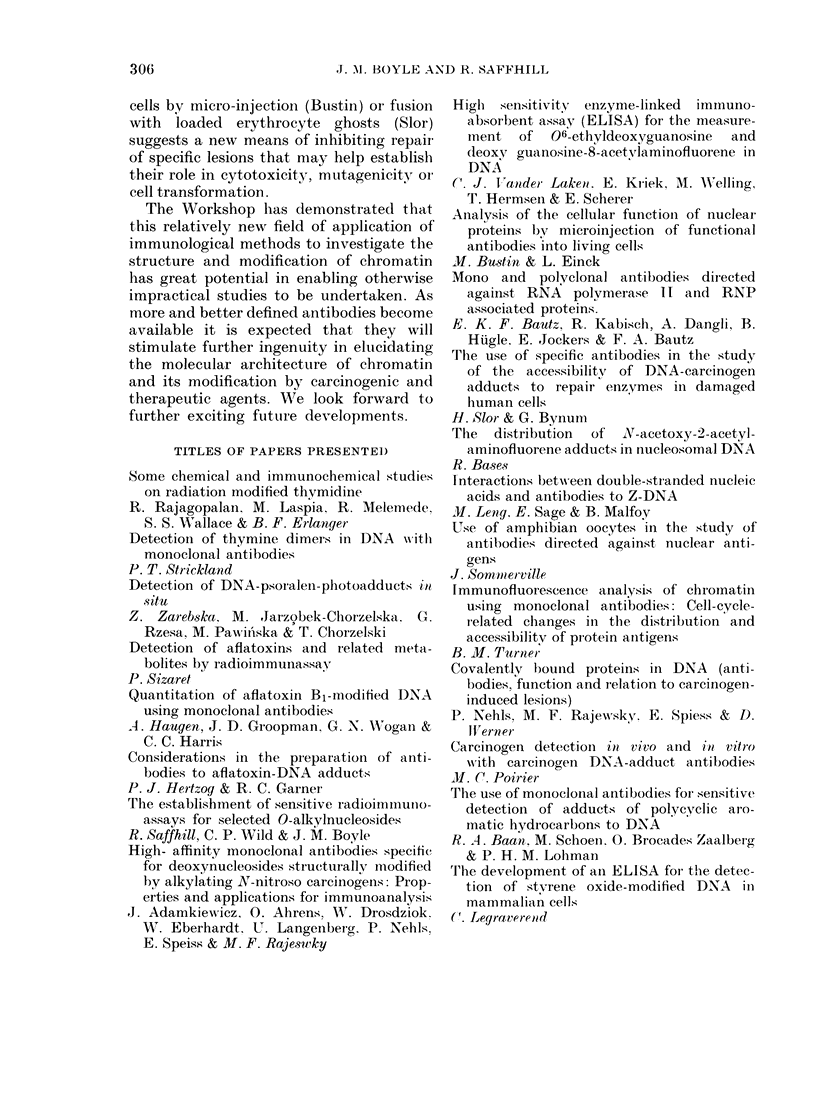# International workshop on immune assay of nuclear antigens relevant to carcinogenesis and chemotherapy

**Published:** 1982-08

**Authors:** 


Br. J. Cancer (1982) 46, 304

### INTERNATIONAL WORKSHOP ON IMMUNE ASSAY OF NUCLEAR ANTIGENS RELEVANT TO CARCINOGENESIS AND CHEMOTHERAPY

### SPONSORED BY BRITISH ASSOCIATION FOR CANCER RESEARCH AND CANCER RESEARCH CAMPAIGN

*Held at the Paterson Laboratories, Christie Hospital and Holt Radium Institute, Manchester M20 9BX*

Convened by J. M. BOYLE AND R. SAFFHILL

26–27 April 1982

IN RECENT YEARS immunological methods have been applied to elucidating the structure and molecular biology of chromatin, and quantifying carcinogen adducts formed in DNA. Such studies have increased the limits of detection previously available and have opened up new avenues for the study of the distribution of adducts in chromatin and within cellular subpopulations. These achievements have accrued from the application of sensitive immunoassays, using polyclonal and now, with increasing importance, monoclonal antibodies. These methods were the subject of a recent International Workshop held at the Paterson Laboratories.

Single-stranded nucleic acids and nucleosides modified by carcinogenic agents, when conjugated either electrostatically or covalently with carrier proteins such as bovine serum albumin or haemocyanin, are able to elicit an antibody response. Such conjugates have been used to produce both polyclonal and monoclonal antibodies specific for DNA damage by both physical and chemical agents. Many antibodies have now been characterized and used to quantify carcinogen-DNA adducts, by radioimmunoassay (RIA), enzyme-linked immunosorbent assay (ELISA), ultrasensitive enzyme radioimmune assay (USERIA) or its fluorometric equivalent using 4-methylumbelliferyl phosphate.

Antibodies were described that are specific for thymine glycol produced in DNA irradiated with ionizing radiation or UV (Erlanger), for thymine dimers which are the major UV photoproduct in DNA (Strickland) and for DNA exposed to UV radiation in the presence of 8-methoxypsoralen (Zarebska). The exquisite specificity possible with antibodies was well demonstrated by monoclonal antibodies specific for $O^6$-alkyldeoxyguanosines, $O^2$-alkyldeoxythymidines and $O^4$-alkyldeoxythymidines (Saffhill, Rajewsky) and by the best polyclonal antibodies produced in rabbits, which were of comparable affinity and specificity (Van der Laken). These antibodies show $>10^7$-fold less reactivity with unmodified nucleosides than with the specific alkylating deoxynucleoside. The primary site of recognition is alkylation at the appropriate $O$-atom and methyl, ethyl and butyl groups are distinguished. Polyclonal and monoclonal antibodies recognizing DNA modified by aflatoxin $B_1$ ($AFB_1$) (Haugen, Hertzog), 2-acetylaminofluorene (AAF) (Poirier, Leng, Van der Laken, Baan), benz(a)-pyrenediolepoxide (BPDE-I) (Slor) and styrene oxide (Legraverend) were also described. Antibodies against $AFB_1$ itself are able to measure $AFB_1$ metabolites in urine (Sizaret).

The sensitivity of the immunoassays is of 0·1–100 fmol, and their usefulness has been demonstrated by the measurement of adducts formed in DNA with biologically meaningful doses. In several cases validation of the immunoassay has been made by demonstrating good statistical agreement with an independent parallel assessment of adduct formation in DNA from cells or tissues exposed to radiolabelled agent.

The reaction with carcinogens is not uniformly distributed along the DNA, and nucleosomal-core proteins may influence the reactivity of *N*-acetoxy-AAF with internucleosomal DNA (Bases). Spacer proteins covalently bound to 3′ and 5′ ends of DNA are believed to form staggered protein discontinuities in DNA that are resistant to alkali and proteases (Werner). Antibodies that recognize these spacers also bind to filaments in the cytoplasm that are not composed of tubulin or actin. Evidence was presented that alkylation adducts may be more prevalent in regions close to the spacers. Reaction of AAF or chlorodiethyl triamine platinum-II chloride with G-C rich DNA-like polymers produces a transition from B-form DNA to Z-form DNA, whilst this power transition is prevented by binding *cis*-diammine dichloroplatinum II (*cis*-platin (Leng). Antibodies specific for Z-DNA were described, which bind to polytene chromosomes of Drosophila, then identifying the natural occurrence of Z-DNA, though whether this occurs in polytene bands or interbands in different species remains controversial.

The structure of chromatin has been probed using antibodies to DNA and chromosomal proteins. Micro-injection of affinity-purified antibodies against HMG and histone proteins into the cytoplasm and nucleus of cells, reveals the distribution of specific antigens and permits the investigation of their role in transcription (Bustin) and embryogenesis (Sommerville). Antibodies specific for different histones have been obtained by immunizing with synthetic hexapeptides of defined sequence (Van Regenmortel). Such probes reveal that the major antigenic sites of histones are at (H3), or close to (H4) the C-terminus, and that these sites are accessible in nucleosomes and core particles. Changes in immunofluorescence associated with two nuclear antigens that are expressed in G1 and G2 but not in S cells were described (Turner). One antigen was expressed in metaphase chromosomes and the other was associated with the nuclear membrane. Transcription of polytene chromosomes is being investigated using monoclonal antibodies against chromosomal proteins (Bautz). One antibody appears specific for RNA polymerase II, whilst another selectively binds heat-shock puffs 93D in *D. melanogaster* and 48BC in *D. hydei*, evidently identifying an hn-RNP class that is stored in the nucleus. Other antibodies recognize proteins that may link hn-RNA to the nuclear matrix.

A major potential of antibodies is their use as histological stains in combination with a second, labelled antibody, to monitor the distribution and repair of adducts in cellular subpopulations of target tissues (Strickland, Zarebska) a method that may prove quantitative if combined with image intensification and analysis (Rajewsky, Bustin). However, the accessibility of DNA *in situ* to antibodies is affected by the degree of packing in the higher structures of chromatin or chromosomes (Nehls). Furthermore the accessibility of adducts in chromatin *in situ* is reflected by the kinetics of excision repair (Slor).

To date, most DNA adducts for which antibodies are available result from the reaction of monofunctional agents. Such antibodies could find immediate application in monitoring individual exposure to specific carcinogens (Legraverend). There is also considerable interest in the possibility of producing antibodies specific for DNA–DNA and DNA–protein cross-linkages which would be of value in monitoring therapy involving bifunctional agents. In some disease conditions it may be useful to measure cell-proliferative status with antinucleoside antibodies which react strongly with cells in S (Bases) or with antibodies to chromatin proteins expressed in G1 and G2 (Turner). The ability to introduce nuclear antibodies into living cells by micro-injection (Bustin) or fusion with loaded erythrocyte ghosts (Slor) suggests a new means of inhibiting repair of specific lesions that may help establish their role in cytotoxicity, mutagenicity or cell transformation.

The Workshop has demonstrated that this relatively new field of application of immunological methods to investigate the structure and modification of chromatin has great potential in enabling otherwise impractical studies to be undertaken. As more and better defined antibodies become available it is expected that they will stimulate further ingenuity in elucidating the molecular architecture of chromatin and its modification by carcinogenic and therapeutic agents. We look forward to further exciting future developments.

TITLES OF PAPERS PRESENTED

Some chemical and immunochemical studies on radiation modified thymidine
R. Rajagopalan, M. Laspia, R. Melemede, S. S. Wallace & *B. F. Erlanger*

Detection of thymine dimers in DNA with monoclonal antibodies
*P. T. Strickland*

Detection of DNA-psoralen-photoadducts *in situ*
*Z. Zarebska*, M. Jarzobek-Chorzelska, G. Rzesa, M. Pawińska & T. Chorzelski

Detection of aflatoxins and related metabolites by radioimmunassay
*P. Sizaret*

Quantitation of aflatoxin $B_1$-modified DNA using monoclonal antibodies
*A. Haugen*, J. D. Groopman, G. N. Wogan & C. C. Harris

Considerations in the preparation of antibodies to aflatoxin-DNA adducts
*P. J. Hertzog* & R. C. Garner

The establishment of sensitive radioimmunoassays for selected *O*-alkylnucleosides
*R. Saffhill*, C. P. Wild & J. M. Boyle

High- affinity monoclonal antibodies specific for deoxynucleosides structurally modified by alkylating *N*-nitroso carcinogens: Properties and applications for immunoanalysis
J. Adamkiewicz, O. Ahrens, W. Drosdziok, W. Eberhardt, U. Langenberg, P. Nehls, E. Speiss & *M. F. Rajeswky*

High sensitivity enzyme-linked immunoabsorbent assay (ELISA) for the measurement of $O^6$-ethyldeoxyguanosine and deoxy guanosine-8-acetylaminofluorene in DNA

*C. J. Vander Laken*, E. Kriek, M. Welling, T. Hermsen & E. Scherer

Analysis of the cellular function of nuclear proteins by microinjection of functional antibodies into living cells
*M. Bustin* & L. Einck

Mono and polyclonal antibodies directed against RNA polymerase II and RNP associated proteins.
*E. K. F. Bautz*, R. Kabisch, A. Dangli, B. Hügle, E. Jockers & F. A. Bautz

The use of specific antibodies in the study of the accessibility of DNA-carcinogen adducts to repair enzymes in damaged human cells
*H. Slor* & G. Bynum

The distribution of *N*-acetoxy-2-acetylaminofluorene adducts in nucleosomal DNA
*R. Bases*

Interactions between double-stranded nucleic acids and antibodies to Z-DNA

*M. Leng*, *E.* Sage & B. Malfoy

Use of amphibian oocytes in the study of antibodies directed against nuclear antigens
*J. Sommerville*

Immunofluorescence analysis of chromatin using monoclonal antibodies: Cell-cycle-related changes in the distribution and accessibility of protein antigens

*B. M. Turner*

Covalently bound proteins in DNA (antibodies, function and relation to carcinogen-induced lesions)
P. Nehls, M. F. Rajewsky, E. Spiess & *D. Werner*

Carcinogen detection *in vivo* and *in vitro* with carcinogen DNA-adduct antibodies

*M. C. Poirier*

The use of monoclonal antibodies for sensitive detection of adducts of polycyclic aromatic hydrocarbons to DNA
*R. A. Baan*, M. Schoen, O. Brocades Zaalberg & P. H. M. Lohman

The development of an ELISA for the detection of styrene oxide-modified DNA in mammalian cells
*C. Legraverend*